# Phenotypic relationships between heart score and feed efficiency, carcass, and pulmonary arterial pressure traits^1^

**DOI:** 10.1093/tas/txaa114

**Published:** 2020-12-22

**Authors:** Kathryn R Heffernan, Milton G Thomas, Richard M Enns, Timothy Holt, Scott E Speidel

**Affiliations:** 1 Department of Animal Sciences, Colorado State University, Fort Collins, CO; 2 Department of Clinical Sciences, Colorado State University, Fort Collins, CO

## INTRODUCTION

**Figure 1.**
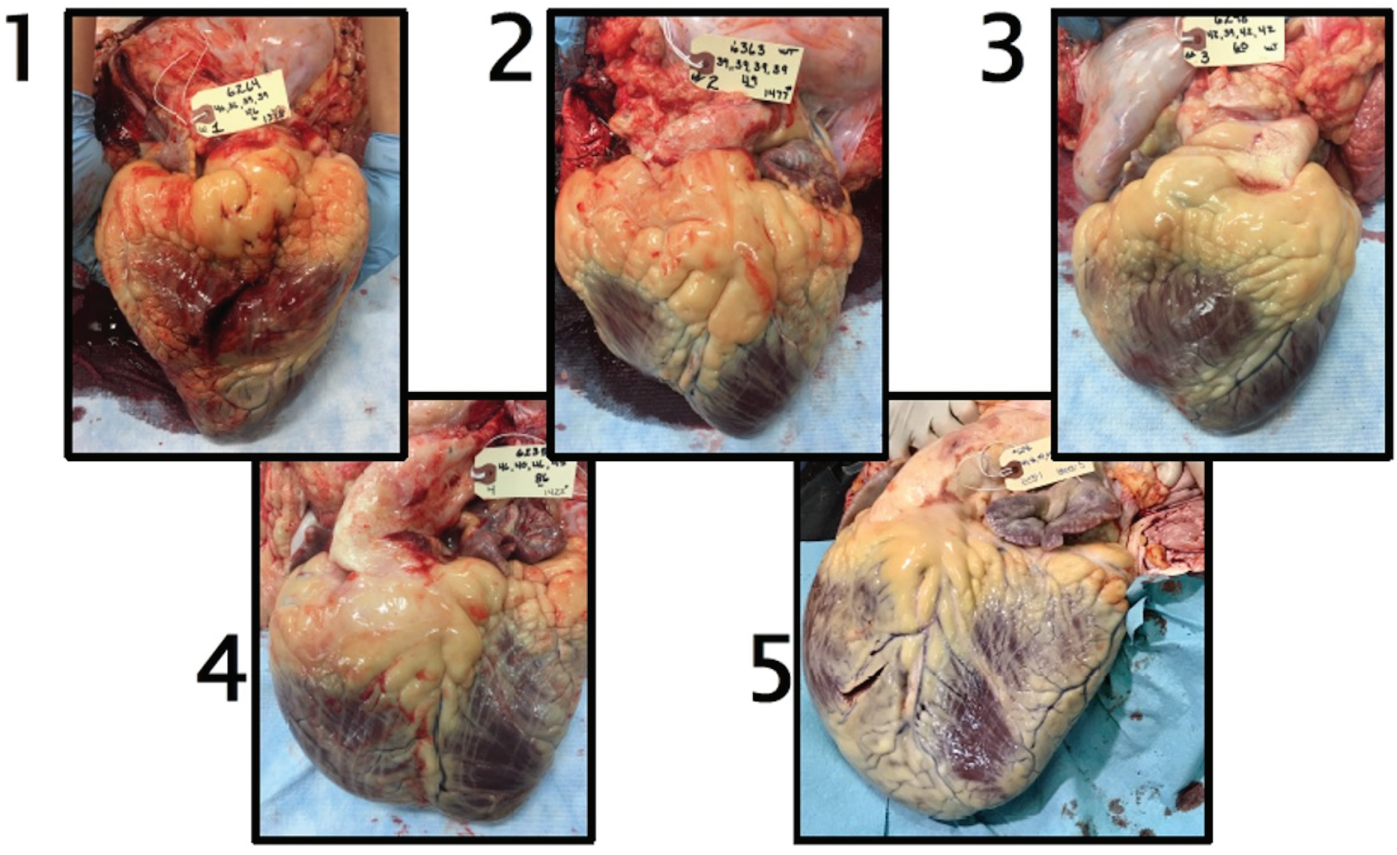
Photographs of typical beef cattle hearts.

Feedlot heart disease (FHD) is a cardiopulmonary condition observed in fed cattle at elevations between 800 and 1,600 m (Neary et al., 2015). Feedlot heart disease results in premature mortality as a result of right-sided heart failure stemming from increased pulmonary vessel pressures and cardiopulmonary remodeling, which is also known as pulmonary hypertension (PH) (Krafsur et al., 2019). This is similar to the well-documented high mountain disease (HMD), which is characterized as the progression of PH resulting from chronic exposure to environmental hypoxia at high altitude (Hecht et al., 1962). Pulmonary arterial pressure (PAP) is abnormally high when an animal experiences PH. Increased PAP leads to *cor pulmonale*, where the heart’s right ventricle becomes enlarged, causing increased work to move the blood from an area of lower pressure (i.e., right ventricle) to an area of higher pressure (i.e., pulmonary artery) resulting in excessive muscle contraction which stretches muscle fibers and can end in right-sided heart failure (Thomas et al., 2018). An animal is given a heart score (HS; scale of 1 to 5 with 5 being extreme structural changes in the heart, Fig. 1) at harvest to categorize the degree to which heart remodeling occurred.

There is evidence that FHD has doubled in fed cattle in the last decade and while FHD outcomes are the same as HMD (Neary et al., 2016), FHD may also be a consequence of pathophysiological remodeling of the left ventricle and pulmonary venous circulation (Krafsur et al., 2019) and occurs at a much lower elevation. An indicator of FHD is mean PAP (mPAP), which is a measurement taken during a veterinary procedure by threading a catheter containing a transducer through the jugular vein and right side of the heart in order to measure pressure in the pulmonary artery. Low-risk cattle have mPAP measurements between 34 and 39 mm Hg, moderate-risk ranges from 40 to 45 mm Hg, and high-risk cattle have a mPAP of 46 mm Hg or greater (Holt and Callan, 2007; PAP, 2020). Since knowledge of the causes of FHD is limited, the objective of this study was to evaluate phenotypic relationships of feedlot efficiency traits, carcass traits, and PAP so we can begin to understand the biological relationships between fed cattle performance and PH.

## MATERIALS AND METHODS

### Data Collection and Editing

The Institutional Animal Care and Use Committee at Colorado State University (approval number 18-8367A) approved all procedures. Mean pulmonary arterial pressure, feed efficiency phenotypes, and carcass data were collected on 89 Black Angus steers from the 2017 calf crop of the Colorado State University’s Beef Improvement Center (BIC) located in Saratoga, Wyoming at an elevation of approximately 2,165 m. These steers were all born in the spring, beginning in March and ending in June. All individual animals were weaned on the 3rd of the October in 2017 and a portion (*n* = 16) were castrated in the same month. These steers were PAP tested on two separate days of the following year in 2018. A majority were tested on the 5th of January as steers for PAP. Subsequently, in early January of 2018, these steers were shipped to the Feed Intake Unit (FIU; Grow Safe System) at the Agricultural Research, Development and Education Center (ARDEC) in Fort Collins, CO. Steers were housed in the FIU for a 21-d warm-up period, followed by a 70-d efficiency test and within that time, feed efficiency phenotypes were collected. While in the FIU, the steers that had PAP measurements taken on the 5th of January were evaluated for average daily gain (ADG), total gain (GAIN) over those 70 d, average dry matter intake (AVG DMI), and average feed to gain ratio (AVG F:G). The rest of the animals that were not sent to the FIU were tested on the 20th of March for a yearling bull PAP and subsequently castrated and shipped to ARDEC as they did not meet criteria for sale as yearling breeding bulls. After those 90 d in the FIU, all 89 steers were then sent to the Easter Colorado Research Center in Akron, Colorado, for finishing.

Cattle were finished to a perceived visual grade of choice. The steers were then shipped as a group to a harvesting plant in Fort Morgan, Colorado (Cargill Meat Solutions) in December of 2018. Heart scores were given immediately postharvest, as per a scoring system developed by Dr. Tim Holt at Colorado State University. In this scoring system, HSs ranged from 1 to 5, where 1 represents a physiologically normal heart and 5 represents a heart that has undergone significant remodeling, especially the right ventricle (Fig. 1). Typically, a HS of 5 is not observed postharvest as these individuals typically do not survive through the fattening phase. Carcass data were collected following a 24-h chilling period. The carcass traits evaluated in this study consisted of Hot Carcass Weight (HCW), Marbling (MARB), Preliminary Yield Grade (PYG), Back Fat in Inches (FAT), Rib Eye Area (REA), USDA Yield Grad (UYG), and Calculated Yield Grade (CYG).

### Statistical Analysis

Data were analyzed using a multiple linear regression model to evaluate the relationship between HS and each trait. Overall, each individual phenotype was regressed on HS and age using the equation presented below:

$$\begin{array}{l} y_{i}=\;\mu +B_{1}x_{1}+\;B_{2}x_{2}+e_{i} \end{array}$$

where *y*_*i*_ is the vector of observed phenotypes for the trait of interest, which included PAP, the carcass traits and the feed efficiency traits, *µ* is the mean of the observations, *B*_1_ is the parameter of the population regression line of the categorical fixed effect of HS, *x*_1_ is the vector of predictor variables for the categorical fixed effect of HS, *B*_2_ is the parameter of the population regression line of the continuous fixed effect of age, *x*_2_ is the vector of predictor variables for the continuous fixed effect of age, and *e*_*i*_ is the vector of random residuals. Least square means were calculated per trait, fitting each individual trait as *y*_*i*_ and using HS on age to predict *y*_*i*_ using the *emmeans* command in the Estimated Marginal Means package in R (R Core Team, 2018). Contrasts between HSs per trait were calculated using the pairwise command, which is also in the Estimated Marginal Means package in R.

## RESULTS AND DISCUSSION

Summary statistics for each trait evaluated are presented in Table 1. While all 89 steers are represented in the carcass traits evaluated, only 73 steers had feed efficiency phenotypes. These 73 steers were the group that was PAP tested on the 5th of January for steer PAP, while the rest of them were PAP tested on the 20th of March after the bull performance test. The arithmetic means in Table 1 were within the range of expectation from previous studies; however, HS overall was a lower average due to the majority of steers (*n* = 37) having a HS of 2, while less (*n* = 5) had a HS of 4 (Fig. 1). The lack of higher HSs could have happened because these steers were progeny of a herd adapted to high altitude; therefore, they were Angus cattle that could accommodate environments with reduced oxygen.

**Table 1.** Summary statistics of PAP, feed efficiency traits, and carcass traits

Trait^1^	Number	Mean	SD	Min	Max
HS	89	1.9	0.867	1.0	4.0
PAP	89	42.6	11.383	35.0	117.0
AVG F:G	73	2.4	0.434	1.5	3.7
PYG	89	3.7	0.394	2.6	4.8
USDA YG	89	3.3	0.723	2.0	5.0
HCW	89	407.3	40.38	240.0	476.3
MARB	89	5.8	0.946	4.1	9.3
FAT	89	17.8	4.013	5.1	27.9
REA	89	87.1	8.503	61.9	105.8
CYG	89	3.7	0.656	2.0	5.0
ADG	73	1.7	0.305	0.7	2.4
GAIN	73	90.6	15.877	38.6	127.0
AVG DMI	73	8.9	1.662	4.4	12.7

^1^HS = heart score; PAP = mean pulmonary arterial pressure (mm Hg); AVG F:G = average feed to gain (kg); PYG = preliminary yield grade; USDA YG = USDA yield grade; HCW = hot carcass weight (kg); MARB = marbling score; FAT = back fat (mm); REA = rib eye area (cm^2^); CYG = calculated yield grade; ADG = average daily gain (kg); GAIN = total gain (kg); AVG DMI = average dry matter intake (kg).

From the statistical model, HS was found to be important for mPAP (*P* < 0.05) and approached importance for AVG DMI and AVG F:G (*P* < 0.10). No other traits were found to be significantly affected by HS. For age, the only traits found to be significantly influenced were HCW and AVG DMI (*P* < 0.05). While mPAP was found to be importantly impacted by HS, the mPAP of cattle with HSs 1, 2, and 3 were similar; however, these three scores individually differed (*P* < 0.0001) with a HS of 4 (Table 2). For AVG DMI, HS approached significance (*P* = 0.0205). The rest of the traits did not have a relationship with HS and did not differ between their respective means (Table 2).

**Table 2.** Least square means followed by their standard error per heart score per trait

HS	PAP^1,2^	AVG F:G^1,3^	PYG^1^	USDA YG^1^	HCW^1^	MARB^1^	FAT^1^	REA^1^	CYG^1^	ADG^1^	GAIN^1^	AVG DMI^1,3^
1	41.40^a^ (1.68)	2.25^a^ (0.08)	3.63^a^ (0.07)	3.27^a^ (0.13)	410.50^a^ (6.99)	5.66^a^ (0.17)	16.51^a^ (0.76)	89.03^a^ (1.48)	3.55^a^ (0.12)	1.81^a^ (0.06)	93.89^a^ (3.17)	8.89^ab^ (0.32)
2	40.50^a^ (1.59)	2.44^a^ (0.07)	3.73^a^ (0.07)	3.37^a^ (0.12)	408.67^a^ (6.62)	5.81^a^ (0.16)	17.53^a^ (0.76)	86.45^a^ (1.42)	3.76^a^ (0.11)	1.76^a^ (0.05)	91.63^a^ (2.88)	9.39^a^ (0.29)
3	41.80^a^ (2.59)	2.21^a^ (0.11)	3.68^a^ (0.11)	3.16^a^ (0.20)	399.16^a^ (10.75)	5.98^a^ (0.26)	17.02^a^ (1.02)	85.16^a^ (2.32)	3.68^a^ (0.18)	1.65^a^ (0.09)	85.73^a^ (4.36)	7.98^b^ (0.43)
4	68.30^b^ (4.33)	2.72^a^ (0.19)	3.73^a^ (0.18)	3.21^a^ (0.33)	396.44^a^ (17.96)	5.64^a^ (0.43)	17.53^a^ (1.78)	84.52^a^ (3.81)	3.75^a^ (0.30)	1.54^a^ (0.14)	79.83^a^ (7.06)	8.75^ab^ (0.70)

^1^PAP = mean pulmonary arterial pressure (mm Hg); AVG F:G = average feed to gain (kg); PYG = preliminary yield grade; USDA YG = USDA yield grade; HCW = hot carcass weight (kg); MARB = marbling score; FAT = back fat (mm); REA = rib eye area (cm^2^); CYG = calculated yield grade; ADG = average daily gain (kg); GAIN = total gain (kg); AVG DMI = average dry matter intake (kg).

^2^HS is significant to the specific trait (*P* < 0.05).

^3^HS is approaching significance with the specific trait (*P* < 0.10).

^ab^Within each column, different superscripts represent statistically significant differences of the means between heart scores for the specific trait (*P* < 0.05).

**Figure 2.**
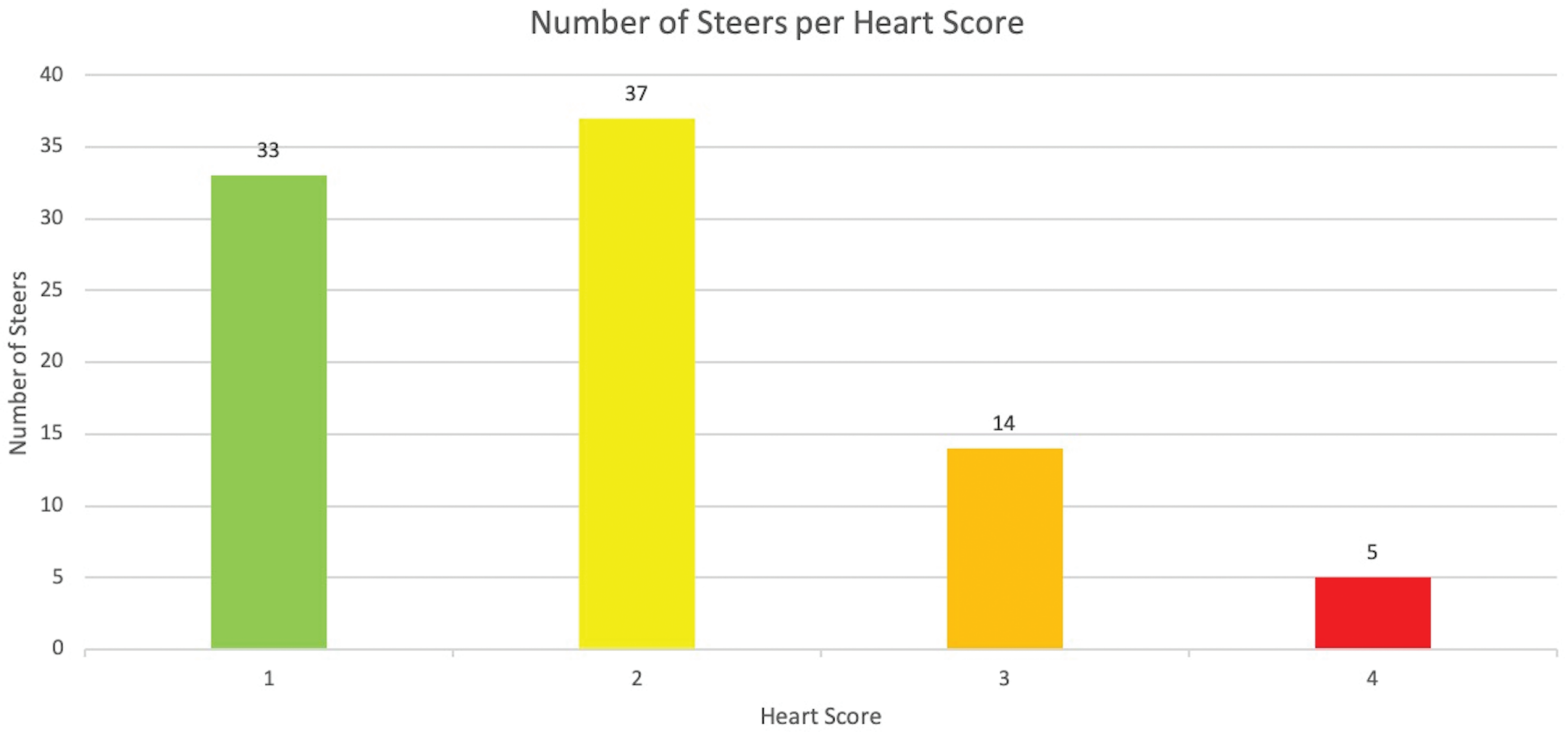
Chart of the number of steers in each HS.

However, while most traits did not show a significant influence from HS, we observed trends within each trait. In general, the standard errors of all of the traits increased as HS increased, which could be due to the small sample size (Table 1) combined with the non-normal distribution of HS (Fig. 2). Pulmonary arterial pressure is consistent for HSs 1 through 3, with an increase between scores 3 and 4 (Table 2). The traits of UYG, PYG, CYG, FAT, and ADG increased between HSs 1 and 2, sharply decreased between 2 and 3, and increased again between 3 and 4 (Table 2). Average feed to gain showed an increase as HS increased, which suggests that animals become less feed efficient as HS increases. Hot carcass weight and REA decreased as HS increased, which could indicate that as HS increases, the carcass quality decreases. Marbling showed an increase between HSs 1 through 3, but then a decrease between 3 and 4, which also showed a possible relationship between HS and carcass quality. Average daily gain and GAIN showed a decrease as HS increased as well, possibly demonstrating that as HS increases, animals tend to gain less. Jennings et al. (2019) reported a similar relationship with partial phenotypic correlations between PAP, HS, and carcass traits adjusting for a finishing system. The authors of that study reported that HS was negatively correlated with HCW, backfat, yield grade, rib eye area, kidney, pelvic, and heart fat, and marbling score. In general, the standard errors of all of the traits increase as HS increases.

In summary, mPAP was positively related to HS. While carcass and feedlot efficiency traits had weak to modest relationships with HS, we observed that as HS increased, feed efficiency and carcass quality tended to decrease. Thomas et al. (2019) reported similar results to this loss of feed efficiency in a study of Angus cattle that had lived their entire life at 1600 m. Specifically, low PAP steers had better (*P* < 0.01) average daily gain and feed to gain ratios than high PAP steers. Further understanding of the causes of FHD and its relationship to mPAP is required to develop effective selection and management practices to reduce the death loss and financial impact FHD has on the cattle feeding industry.

## IMPLICATIONS

While FHD ends in premature mortality, it also causes cattle to be less efficient and decreases carcass quality; all of which will result in significant monetary loss. This disease also occurs at low to moderate elevations, which means that it will affect a wider range of the industry. This will cause more studies to be done on the subject of a possible genetic component to FHD so we can better select against it in the future.



*Conflict of interest statement*. The author(s) declare(s) that there is no conflict of interest.
